# Nasal hyperkeratosis in Griffon breeds: Clinical, histopathological features and the prevalence in the Swedish population compared to a control group and other brachycephalic breeds

**DOI:** 10.1002/vro2.10

**Published:** 2021-05-05

**Authors:** Robert Cikota, Liselotte Åberg, Erika Karlstam, Arman Shokrai, Susanne Åhman

**Affiliations:** ^1^ VetaDerm Göteborg AB Gothenburg Sweden; ^2^ VetaDerm Veterinarklinik Lomma Sweden; ^3^ Department of Pathology National Veterinary Institute of Sweden, SVA Uppsala Sweden

**Keywords:** brachycephalic, Griffon breed, nasal hyperkeratosis

## Abstract

**Background:**

In the Griffon breeds (GB) nasal hyperkeratosis is common and develops already in early adulthood. Breed‐related features and prevalence have not previously been documented.

**Hypothesis/Objectives:**

To describe clinical and histopathological features of nasal hyperkeratosis in GB and to document the prevalence.

**Materials and methods:**

Seven GB dogs with nasal hyperkeratosis were examined. Three histopathological samples were analysed. Owners of 107 GB and 493 control dogs completed a questionnaire distributed via social media.

**Results:**

Typical features of nasal hyperkeratosis in GB included varying degrees of dry, firm, excessive proliferation of keratin, affecting the dorsal or dorsolateral aspect of the planum nasale. Histopathology was characterized by severe, lamellar orthokeratotic and focal parakeratotic hyperkeratosis and multiple small serum lakes. Thirty‐four of 107 GB dogs (31.8%) and 65 of 493 (13.2%) control dogs had varying degree of nasal hyperkeratosis. No sex predisposition was noted. Median age of onset was 3 years for GB, similar to brachycephalic control dogs whereas non‐brachycephalic control dogs had a significantly later age of onset (*p* = 0.0053).

**Conclusions and clinical importance:**

Idiopathic nasal hyperkeratosis is very common in GB dogs and other brachycephalic breeds with nearly one third being affected, often already a young age.

## INTRODUCTION

Idiopathic nasal or nasodigital hyperkeratosis typically occurs in older dogs of various breeds.^1^, ^2^ In spite of being a universally well‐recognized condition, remarkably few peer‐reviewed scientific publications are available.^3^ The only published data on prevalence report 0.4% of dermatology cases and 0.1% of the hospital population having idiopathic nasal or nasodigital hyperkeratosis. The Cocker spaniel breed may be at increased risk.^3^ Brachycephalic breeds are sometimes considered predisposed to nasal hyperkeratosis presumingly due to abnormal anatomy and keratin build‐up; however to our knowledge this has not been well documented.^1^ Other causes of nasal hyperkeratosis in dogs include cutaneous discoid lupus, distemper, ichthyosis, necrolytic migratory erythema, leishmaniosis, pemphigus complex, primary seborrheic dermatitis, systemic lupus erythematosus and zinc‐responsive dermatosis.^1^, ^2^

**FIGURE 1:**
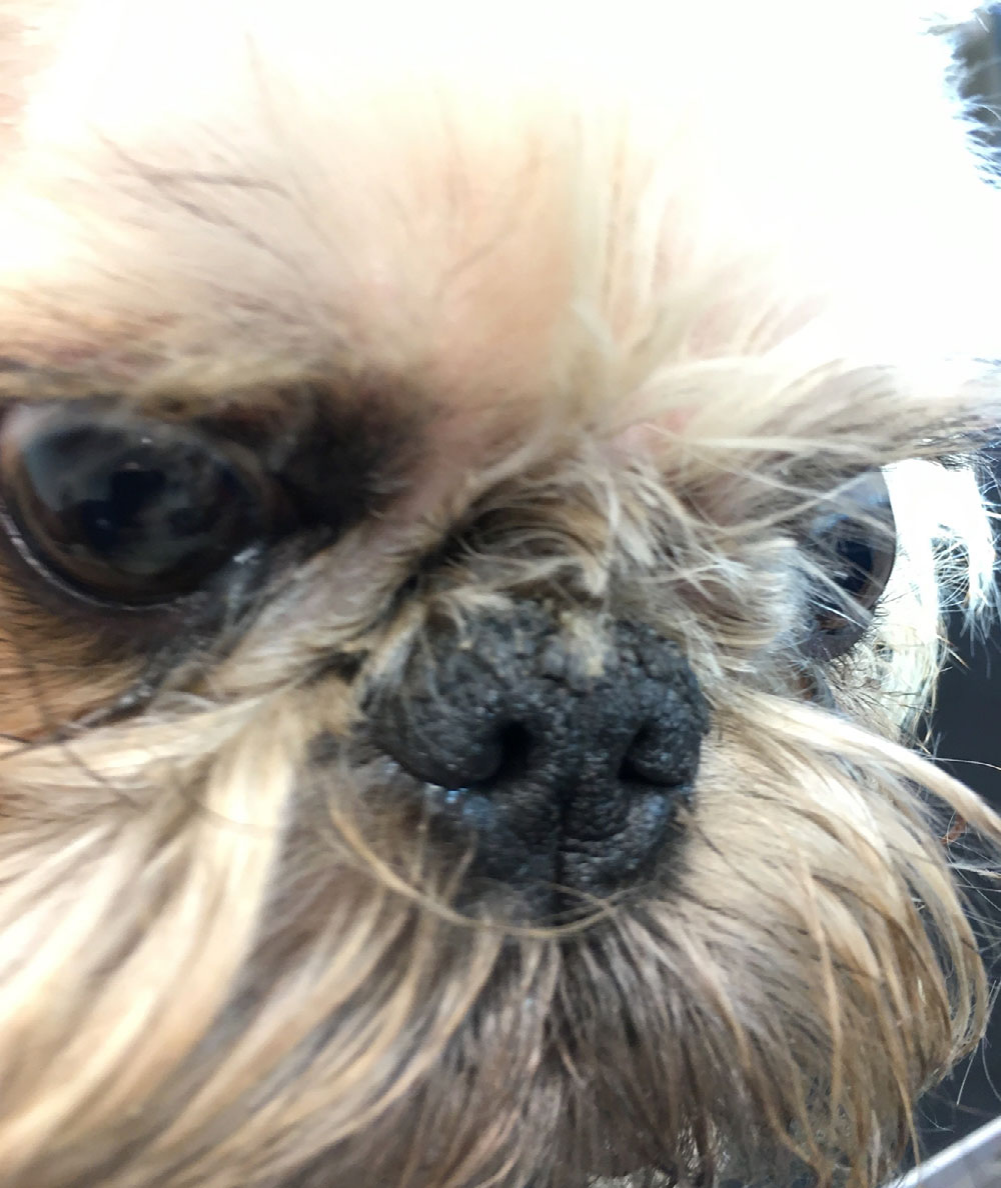
Griffon dog with moderate nasal hyperkeratosis

The Griffon breeds (GB)—Griffon Belge, Griffon Bruxellios and Petit Brabançon—are identical in standards except for coat and colour variants, and they are considered varieties of the same breed and are allowed to mate. In the same litter, all three varieties can be born.^4^, ^5^ GB‐breeders, world‐wide have for decades recognized that, in all other aspects healthy, many dogs have an abnormally dry and firm nose (Figure 1). Owners and breeders refer to this as 'dry nose' and frequently treat it with various over‐the‐counter moisturizers, creams and balms.

The objective of this study was to describe the clinical and histopathological features of idiopathic nasal hyperkeratosis in the GB and to investigate the prevalence among these breeds in Sweden and to compare the prevalence to a control group.

## MATERIALS AND METHODS

### Clinical evaluation

Seven GB dogs with nasal hyperkeratosis were examined by the authors at Vetaderm Veterinärklinik in Lomma and Vetaderm in Kungsbacka, Sweden. One case was presented for nasal hyperkeratosis, and the other six were presented for unrelated reasons, but during the clinical examination nasal hyperkeratosis was observed.

### Questionnaire

A web‐based Google Forms questionnaire was distributed by email to all 140 members of the Swedish Griffon Club and to other Griffon owners via two social media forums on Facebook. For the control group, any breeds, except GB were accepted, and a questionnaire was distributed to five different Facebook‐groups for dog owners to compose the control group. Participation was voluntary with no reimbursement offered, and General Data Protection Regulation (GDPR) was followed regarding collection and storage of data. Owners of healthy dogs with no evidence of nasal lesions were also encouraged to fill out the form in an attempt to minimize selection bias for both groups. The questionnaire was developed to allow owners to self‐evaluate their dog's nose health with respect to clinical signs of nasal hyperkeratosis. Typical photos of a normal nasal planum and of noses with varying degrees of nasal hyperkeratosis were provided with the questionnaire to allow owners to compare their dog´s nose with the images (supplement S1). The questionnaire was submitted online and had 19 questions regarding breed, age, gender, presence and severity of nasal lesions, age at onset, other dermatological signs, general health and any treatment given (Supplement S2 for the complete questionnaire form). The questionnaire was initially piloted with owners of five healthy dogs from a local veterinary practice, and feedback on the questions’ applicability, user‐friendliness and clarity was requested. Further refinement of the questions to aid user‐friendliness was thereafter conducted via review by authors. The online questionnaire was open for 2 months between 1st August and 1st October, and the full dataset was downloaded on 1st October 2019 for the Griffon group and for the control group for 2 months between 27th April and 1st June, and the full dataset was downloaded on 1st June 2020.

### Histopathological samples

Three samples from GB dogs with nasal hyperkeratosis were analysed. Two biopsies were taken post‐mortem from dogs euthanized for unrelated causes with the permission from the owners. One sample was identified after a request to the Swedish Dermatology Study Group for sharing known cases of nasal hyperkeratosis in GB and had been taken as a part of the dermatological work‐up by the referring vet. A search in two Swedish histopathology laboratories data bases did not reveal further samples. All biopsies were fixed in formaldehyde, routinely processed, and embedded in paraffin. Five micrometer thick sections were prepared and stained with haematoxylin and eosin (HE‐stain) and periodic acid‐schiff (PAS‐stain). All biopsy specimens were submitted to National Veterinary Institute of Sweden, SVA. Each slide was evaluated by two pathologists with a special interest in dermatopathology, one being Diplomate European College of Veterinary Pathologists. Referring veterinarians were contacted in order to gather historical, clinical and, if relevant, therapeutic information.

### Statistical procedures

Descriptive statistics were used to summaries the data, which are presented as medians and ranges. The distribution of gender and breeds between groups was compared with the chi‐square test. Statistical analysis was performed using XLSTAT (Addinsoft, 40, rue Damrémont 75018 Paris). Fisher exact test and *t*‐test were used for calculating onset of age and prevalence. A *p*‐value < 0.05 was considered significant.

## RESULTS

### Study population

Seven GB dogs (five Petit Brabançon and two Griffon Bruxellios), aged 2–13 years, four female and three males, were clinically examined. One dog was presented due to severe nasal hyperkeratosis, all others were presented for unrelated conditions (hip luxation, protein losing enteropathy, canine atopic dermatitis and chronic kidney failure), and nasal hyperkeratosis was observed at clinical examination. Three GB dogs (one of each Griffon Bruxellios, Griffon Belge and Petit Brabançon), aged 3,13 and 14 years, two female and one male had nasal biopsies. One had biopsies taken as part of its dermatological diagnostic work‐up, and two were post‐mortem samples. One hundred and seven GB dogs, 52 male and 55 female, were included in the owner survey. The median age was 5 years (range 1–10 years) (Table 1). The control group consisted of 493 dogs of any breed except GB, 222 male and 271 female, and median age was 5 years.

**TABLE 1:** Gender, breed distribution and prevalence of nasal hyperkeratosis

	*n*	Female	Male	Nasal hyperkeratosis	Unaffected
Griffon Belge	20	10	10	9 (45%)	11
Griffon Bruxellios	21	5	16	4 (19%)	17
Petit Brabancon	66	37	29	21 (32%)	45
*Total*	107	55	52	34 (31.8%)	73

### Clinical findings

All seven GB dogs with nasal hyperkeratosis had similar clinical signs, albeit of varying severity. The dorsal or dorsolateral part of the planum nasale was firm and non‐pliable with varying amounts of thick brownish to greyish adherent, dry and rough keratin material accumulating in an uneven way, creating multiple adherent crusts. Occasionally fissures, small erosions and mild to moderate depigmentation were present. The rostral part of nasal planum and nostrils were typically unaffected with normal cobblestone structure. None of the affected dogs had paw pad hyperkeratosis.

### Histopathological findings

**FIGURE 2:**
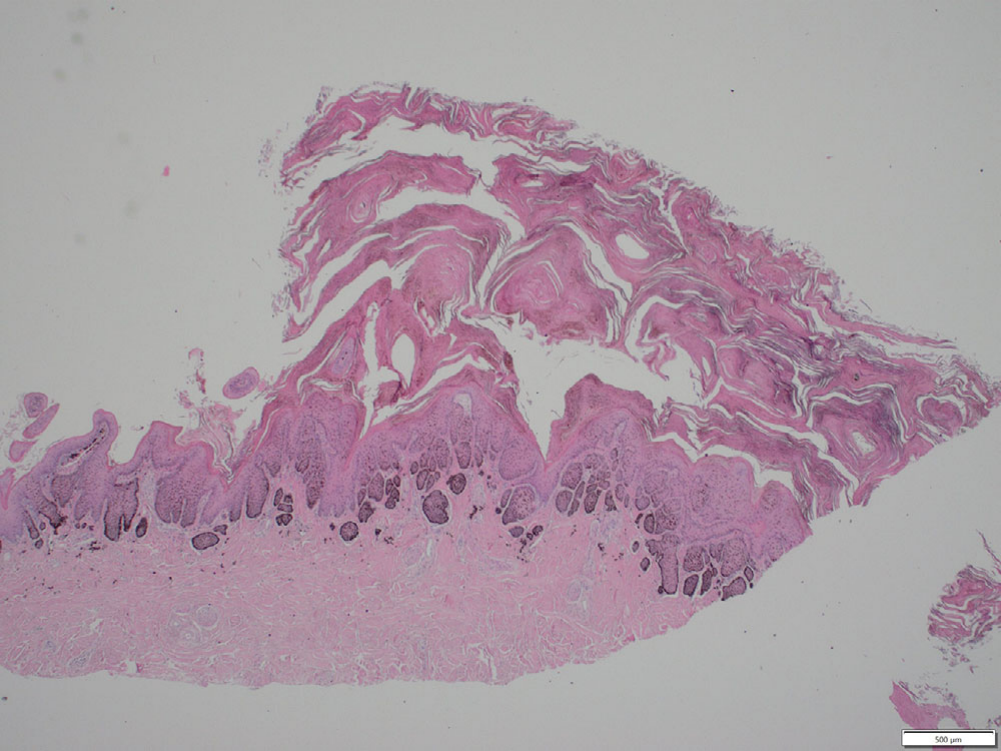
Nasal planum. Epidermis displays moderate acanthosis and moderate to severe orthokeratotic hyperkeratosis. HE‐stain, 10 magnification

**FIGURE 3:**
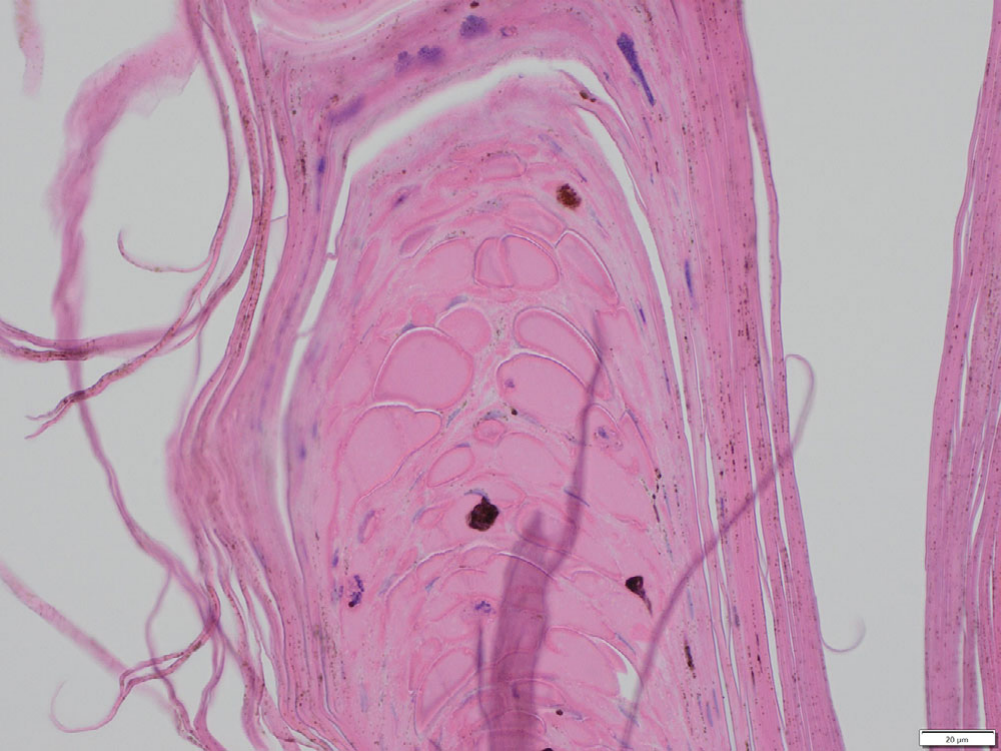
Nasal planum. Numerous serum vesicles are seen within the keratin layer. HE‐stain, 40 magnfication

Similar changes were observed in all biopsies from the three dogs. The epidermis displayed moderate acanthosis and severe, lamellar orthokeratotic hyperkeratosis and in two dogs also focal parakeratosis (Figure 2). In all dogs, small serum lakes were seen multifocally within the keratin layer (Figure 3). Furthermore, in two dogs small amounts of cellular debris and degenerated neutrophils were present multifocally within the keratin layer.

Dermal changes were mild and superficial. Sparse perivascular infiltration of lymphocytes and occasional plasma cells was present. Additionally, in two samples, mild pigmentary incontinence was observed in the superficial parts of the dermis. No changes were found in the deeper dermis.

### Questionnaire

Owners of 107 GB dogs completed the survey. A total of 58 answers came from GB owners in the Swedish Griffon club (corresponding to a response rate of 41%) and 49 from social media forums related to the GB on Facebook. Thirty‐four dogs (31.8%) had lesions that corresponded to nasal hyperkeratosis, confidence interval 23–41% (CI 95%). There was no gender predisposition (*p* = .53). There was no significant difference between the prevalence of nasal hyperkeratosis between the three GB (*p* = .20). The median age of onset was 3 years, range 1–10 years. For the control group, owners of 493 dogs completed the survey. In the control group, 65 dogs (13.2%) had nasal hyperkeratotic lesions. The median age of onset for the complete control group was 3.5 years, and the median age of onset for the subset of brachycephalic dogs in the control group was 2 years. The age of onset did not differ between GB and brachycephalic control dogs (*p* = 0.32). The median age of onset in non‐brachycephalic control dogs was 6 years, significantly higher (*p* = 0.0053). There was a significant difference in prevalence between GB and dogs in the complete control group (*p* < 0.00001). In the complete control group, there were 114 different breeds, and the five most frequent breeds were Golden retriever (*n* = 25), German Shepherd (*n* = 35), Labrador retriever (*n* = 21), Portuguese water dog (*n* = 23) and mixed breed (*n* = 62). All other breeds were represented by less than 20 dogs. When the control group was subdivided into brachycephalic and non‐brachycephalic dogs, a total of 40 brachycephalic, non‐GB dogs were identified, and among these the reported prevalence of nasal hyperkeratosis was 22 of 40 (55%) and among the non‐brachycephalic dogs 43 of 453 (9.5%).

Owners to 16 of the 34 GB dogs provided digital photos of their dogs, confirming visually typical nasal hyperkeratosis. Owners to 26 of 34 owners to Griffon dogs reported that they treated the dogs with over‐the‐counter creams or moisturizers, and 24 reported partial to complete improvement. In the control group, dogs with nasal hyperkeratosis, 40 owners reported that they had treated the dogs with over‐the counter creams or moisturizers, and 31 reported partial to complete improvement. In 15 cases the owner of GB dogs reported the severity of the nasal hyperkeratosis to vary over time.

## DISCUSSION

Canine nasal hyperkeratosis is the result of an abnormal cornification process. It may reflect either an excessive proliferation of keratinocytes, an abnormal keratinocyte‐retention, a lack of desquamation or a combination thereof. The normal shiny and pliable canine planum nasale becomes dry, firm, rough and hyperplastic. Nasal hyperkeratosis may be senile or may develop secondary to a large variety of diseases, including discoid lupus erythematosus, pemphigus foliaceus, zinc‐responsive dermatosis, cutaneous lymphoma and leishmaniasis.^1^ Idiopathic nasal hyperkeratosis in otherwise healthy dogs has been described, most commonly in geriatric dogs of different breeds and may also be associated with concurrent footpad hyperkeratosis.^1^, ^3^

In the GB, the clinical appearance is typically similar to that of idiopathic nasal hyperkeratosis. However, the relatively young age of onset and a high proportion of affected animals within the breed suggest either a connection to the anatomical features or a different genetic component being present. Brachycephalic breeds are often considered predisposed to nasal hyperkeratosis presuming due to abnormal anatomy and keratin build‐up; however to our knowledge, this has not been well documented.^1^ In a recently published treatment study of nasal hyperkeratosis, the study group mainly consisted of brachycephalic breeds but due to lack of publications with a control group, the connection between brachycephalic anatomy and nasal hyperkeratosis remains anecdotal.^6^

The original purpose of this study was to document the suspected high prevalence and the young age of onset of nasal hyperkeratosis in the GB. However, when analysing the control group data, it became evident that neither the young age of onset nor the high prevalence was unique to the GB, but rather a feature of all brachycephalic dogs. To our knowledge this is the first study reporting the prevalence of nasal hyperkeratosis in brachycephalic dogs compared to a larger control group.

The pathogenesis of nasal hyperkeratosis in brachycephalic breeds is still poorly understood. For example, it is still unknown whether the severity of nasal hyperkeratosis differs between various phenotypes of the nose itself such as absolute nose length. The owner survey was not designed to allow the owner to grade nasal lesions for severity neither to classify nasal phenotype or length.

In Irish terriers and Kromfohrländer, a genetic background to footpad hyperkeratosis has been documented, and an inherited monogenic autosomal recessive mutation in the FAM83G gene has been described.^7^, ^8^ A common approach for identifying canine genetic risk factors has been to use SNP‐based genotyping and genome‐wide association study (GWAS).^7^, ^9^ For autosomal recessive diseases caused by a single gene mutation, a sample collection of 10–20 affected dogs and 10–20 healthy dogs is needed.^9^ In two studies of hereditary footpad hyperkeratosis, the mutated gene was identified using only 13 affected and 29 healthy Kromfohrländer dogs and 10 affected and 21 healthy Irish terriers, respectively.^7^ A GWAS comparing non‐affected and affected GB dogs would be desirable to identify if nasal hyperkeratosis in GB is an inherited disease as in other breeds with familiar hyperkeratosis, and what gene(s) may be responsible or if the hyperkeratosis may be directly or indirectly associated with brachycephalic genes such as SMOC2 and BMP3 gene.^10^, ^11^

The main histopathology findings were severe lamellar orthokeratotic hyperkeratosis with small serum vesicles in the keratin layers. The focal parakeratotic hyperkeratosis and accumulation of degenerated neutrophils within the keratin layers were considered secondary changes. The stratum corneum in a normal planum nasale is invariably compact and generally orthokeratotic in nature; however a third of normal dogs will have focal areas of parakeratosis in the absence of inflammation.^12^

Small serum vesicles are not present in the normal planum nasale but may develop in any hyperkeratotic nose. Based on two studies, small intracorneal vacuoles (serum lakes) are a common finding in many hyperkeratotic and parakeratotic skin diseases of the dog. In hereditary nasal parakeratosis of the Labrador Retriever, both larger and more frequent serum lakes are characteristically present compared to specimens from dogs with other hyperkeratotic or parakeratotic diseases.^13^, ^14^

The histopathological analysis is limited by the small sample, and the fact that two of the three dogs were old and euthanized due to other diseases. The concurrent disease and the old age may have affected the histopathology result. However, both dogs had, according to the owners, similar lesions for several years. A large sample of nasal biopsies from GB dogs including both affected and non‐affected animals would be desirable to establish histopathology. Because affected dogs are usually asymptomatic or experience only mild discomfort, we considered taking biopsies from these dogs unethical and therefore decided not to apply for ethical permission.

In the owner survey for GB dogs, 31.8% of the dogs were reported to have nasal hyperkeratosis. There may be a risk of a proportionally higher participation by owners of affected individuals in a voluntary non‐randomized study, which would result in a falsely high estimation of the prevalence. In an attempt to decrease selection bias, owners to healthy GB dogs were actively encouraged to submit the questionnaire for their healthy pets. The same selection bias is suspected in the control group and may even be more pronounced, mainly because we did not have an opportunity to contact responders about the importance of reporting also non‐affected individuals. However, it seems to be a genuinely common condition in the GB in spite of the previous lack of documentation in the literature. In the US there is even a special nose balm, called 'Brussels Griffon Nose Butter' on the market. In this survey a majority (76,5%) of the owners of GB dogs with nasal hyperkeratosis reported that they had treated the dogs with over‐the‐counter creams or moisturizers, with variable results. In 15 cases the owner reported the severity of the nasal hyperkeratosis to vary over time, possibly depending on the frequency of bathing, treatment with creams or moisturizers.

A possible weakness of a questionnaire survey is the sample size relative to the entire population. In Sweden, approximately 200 puppies are registered in the GB each year. There are an estimated 2000 GB dogs in Sweden, which makes the sample size equivalent to approximately 5.3% of the breed. The survey was sent via the Swedish Griffon club to 140 members, of which 58 replied (41%). One way of increasing the sample might have been to keep the questionnaire open longer. However, it was evident that the vast majority of replies were received within the first days of sending out the questionnaire for both groups and just a few additional answers after a reminder was sent out. A weakness of any owner survey is self‐evaluation of their animals' disease. In the Griffon survey, owners of 16 of the 34 reported cases with nasal hyperkeratosis provided digital images of their dogs nose that allowed a visual confirmation of the typical clinical features. In the control group, no digital images were provided. The strength of the survey would increase if all dogs were evaluated by a dermatologist.

## CONCLUSION

Nasal hyperkeratosis is a common and usually asymptomatic condition in the GB. Typical clinical features include varying degrees of dry, firm, hyperkeratotic proliferation of keratin affecting the dorsal or dorsolateral aspect of the planum nasale. The median age of onset was 3 years. In a web‐based questionnaire, 31.8% of the GB dogs had nasal hyperkeratosis compared to 13.2% in the control group. Brachycephalic dogs in the control group had a frequency of nasal hyperkeratosis similar to that of GB. Typical histopathological changes in GB dogs were severe lamellar orthokeratotic hyperkeratosis with small serum vesicles within the keratin layers. Further studies are needed to establish if nasal hyperkeratosis in GB and other brachycephalic breeds are associated directly or indirectly with specific genes.

## CONFLICT OF INTEREST

The authors declare that there is no conflict of interest that could be perceived as prejudicing the impartiality of the research reported.

## Supporting information

Supporting Information

Supporting Information

Supporting Information
