# Supplementary material for: Nasal hyperkeratosis in Griffon breeds: Clinical, histopathological features and the prevalence in the Swedish population compared to a control group and other brachycephalic breeds
Source: Vet Rec Open. 2021 May 5;8(1):e10. doi: 10.1002/vro2.10 (PMC8110113; doi:10.1002/vro2.10)
Supplement: Supplementary file 1 — Supporting Information [file VRO2-8-e10-s001.pdf]

Supplement S1:

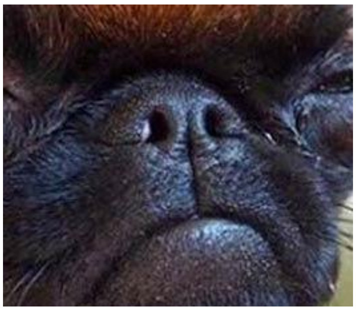

Example-picture of normal Griffon nose.

Supplement S2:

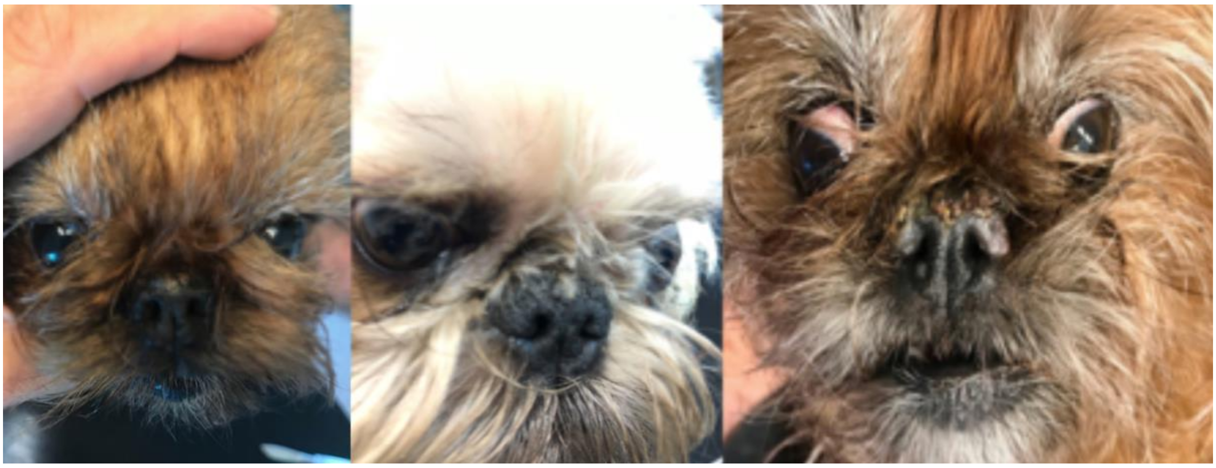

Example-picture of nasal hyperkeratosis (mild, moderate and severe lesions).
