# Supplementary material for: Nasal hyperkeratosis in Griffon breeds: Clinical, histopathological features and the prevalence in the Swedish population compared to a control group and other brachycephalic breeds
Source: Vet Rec Open. 2021 May 5;8(1):e10. doi: 10.1002/vro2.10 (PMC8110113; doi:10.1002/vro2.10)
Supplement: Supplementary file 2 — Supporting Information [file VRO2-8-e10-s003.pdf]

Supplement table 2.

1. Email address?
2. Name?
3. The dog's name?
4. SKK registration number (voluntary information)?
5. What breed is your dog?
6. What gender is your dog?
7. What age is your dog?
8. Does your dog have dry coatings or sore changes on the top of the nose? Sometimes the color of the nose also changes. The pictures show examples of typical mild, moderate and significant changes.
9. At what age did the dog's nose change debut?
10. Have biopsies (tissue samples) been taken from the nose?
11. Does the dog have similar changes on the pads?
12. Is the dog treated for nose changes?
13. Is the dog treated with topical prescription drugs (eg cortisone, antibiotics, Protopic, Propylene glycol) against nose changes?
14. Did the treatment help?
15. Is the dog treated with non-prescription medication (eg vaseline, omega3, emollient ointment) against nasal changes?
16. Did the treatment help?
17. Is the dog treated with other (internal) medicines for nose changes?
18. Did the treatment help?
19. Can we contact you if we have additional questions?

Other comments:
